# Beyond Triage: The Critical Role of Emergency Nurses in COPD Assessment and Management—Insights from Patients and Staff

**DOI:** 10.3390/nursrep16040136

**Published:** 2026-04-14

**Authors:** Clint Moloney, Gavin Beccaria, Amy B. Mullens

**Affiliations:** 1 School of Nursing and Midwifery, University of Southern Queensland, Toowoomba, QLD 4350, Australia; gavin.beccaria@unisq.edu.au (G.B.); amy.mullens@unisq.edu.au (A.B.M.); 2 Institute of Health, University of Southern Queensland, Toowoomba, QLD 4350, Australia; 3 School of Health, Psychological and Medical Sciences, University of Southern Queensland, Toowoomba, QLD 4350, Australia; 4 School of Health, Psychological and Medical Sciences, University of Southern Queensland, Ipswich, QLD 4305, Australia

**Keywords:** COPD, emergency department, nursing care, phenomenology, qualitative research, care coordination, patient education, obstructive sleep apnoea, transitional care

## Abstract

**Background**: Chronic Obstructive Pulmonary Disease (COPD) remains a leading cause of emergency department (ED) presentation, hospitalisation, and preventable healthcare utilisation worldwide. Although guidelines advocate coordinated, preventative, and community-based management, care within ED settings often remains reactive and crisis-driven. Nurses occupy a central role in COPD management; however, the experiential dimensions of nursing practice and its contribution to improving patient outcomes are insufficiently understood. **Objectives**: To explore the lived experiences of patients, nurses and medical officers regarding COPD presentations to the ED, with particular focus on the nursing role in assessment, coordination, education, and identification of unmet and comorbid care needs. **Methods**: A qualitative phenomenological approach was undertaken across three regional Australian EDs. Purposive sampling recruited patients presenting with acute exacerbations of COPD and nursing and medical officers involved in their care. Semi-structured interviews were conducted and transcribed verbatim. Data were analysed using Braun and Clarke’s thematic analysis framework, supported by reflexive discussion and audit trails to enhance rigour. **Results**: Six interrelated themes were identified: (1) nursing within a “crisis first” model of care; (2) holistic assessment and translation of complexity; (3) education and care coordination as preventative nursing work; (4) relational care and therapeutic connection; (5) nurses as sentinels for undiagnosed comorbidities, particularly obstructive sleep apnoea; and (6) system pressures constraining optimal nursing practice. Participants consistently described nurses as the clinicians who stabilised acute episodes, interpreted contextual risks, coordinated services, and provided relational and educational support, yet whose preventative contributions were limited by time and organisational demands. **Conclusions**: ED nurses function as critical integrators between acute stabilisation and chronic disease management for patients with COPD. Formalising nurse-led assessment, education, coordination, and sleep-disordered breathing screening may reduce avoidable ED presentations and enhance patient-centred outcomes. Investment in structured nursing models represents a key opportunity for improving COPD care delivery.

## 1. Introduction

Chronic Obstructive Pulmonary Disease (COPD) is a progressive respiratory illness associated with substantial morbidity, mortality, and escalating healthcare utilisation worldwide. More than 250 million people live with COPD globally, and the condition remains a leading cause of preventable death and hospitalisation, accounting for approximately three million deaths annually [1,2]. In Australia, COPD is consistently among the most common causes of emergency department (ED) presentation and admission in older adults, with acute exacerbations contributing disproportionately to healthcare costs and bed occupancy [3]. Importantly, many exacerbations and subsequent ED visits are considered potentially preventable through earlier recognition of deterioration, coordinated chronic disease management, and structured patient education [3,4,5].

International and national guidelines, including the COPD-X Plan and the Global Initiative for Chronic Obstructive Lung Disease (GOLD), recommend proactive, multidisciplinary, and community-integrated models of care to reduce avoidable admissions [4,5]. The updated Australian COPD-X guidelines further reinforce the importance of coordinated care, early intervention, and structured follow-up in reducing exacerbation burden and hospital utilisation [5]. Within these models, nurses are central to operationalizing evidence-based care, particularly through patient education, early recognition of deterioration, care coordination, and facilitation of continuity across settings. Evidence demonstrates that nurse-led and case-management interventions improve self-management, enhance continuity, and reduce unscheduled healthcare use. Transitional care programmes delivered by nurses have shown reductions in readmissions and improved quality of life among patients with COPD [6], while community respiratory services and coordinated follow-up have been associated with significant reductions in emergency presentations [7]. Despite these advances, recurrent ED attendance remains common, suggesting ongoing fragmentation between hospital and primary care services and variability in adherence to evidence-based COPD management in emergency settings [8,9,10].

Qualitative studies highlight that patients with COPD frequently experience discharge vulnerability, inadequate education, and poor communication across settings, resulting in crisis-driven service use rather than planned care [8,11,12]. Nurses occupy a central role in addressing these gaps, functioning not only as providers of clinical interventions but also as coordinators, educators, and patient advocates within complex and time-pressured ED environments. Within the ED, nurses require competence in advanced respiratory assessment, oxygen titration, medication administration, discharge planning, and referral coordination, often acting as the primary point of continuity for patients across the episode of care [10,13,14].

Beyond technical functions, the nursing role encompasses relational, interpretive, and anticipatory dimensions that are critical to patient outcomes. Person-centred care—including ensuring patients feel recognised, supported, and not alone—has been identified as a defining feature of effective ED nursing practice [15]. Nurses also engage in continuous clinical judgement, balancing competing priorities, managing uncertainty, and responding to rapidly evolving patient conditions. Contemporary evidence highlights the complexity of these roles, including the emotional labour, ethical responsibility, and professional accountability associated with delivering care in high-acuity environments [13,16,17,18]. Furthermore, ED nurses are increasingly expected to enact advanced roles, including clinical leadership, coordination of care pathways, and adherence to evidence-based protocols, such as COPD bundles of care, which have demonstrated variable implementation in practice [9,10]. Despite this, how nurses integrate these responsibilities in real-time clinical decision-making, particularly in COPD presentations, remains underexplored.

Emerging evidence also suggests that undiagnosed comorbidities, particularly obstructive sleep apnoea (OSA), may exacerbate COPD instability. COPD–OSA overlap syndrome is associated with recurrent exacerbations, hypoxaemia, and increased hospital utilisation, yet screening remains inconsistent despite the availability of simple tools such as STOP-Bang [19,20,21]. Nurses, through routine assessment, clinical surveillance, and sustained patient interaction, are uniquely positioned to identify these risks, initiate referral pathways, and contribute to early intervention strategies.

Understanding how nurses and patients experience COPD presentations within the ED is therefore essential to inform sustainable, nurse-led improvements in care delivery. Accordingly, this study aimed to explore the lived experiences of patients, nurses and medical officers to better understand the nursing contribution to assessment, coordination, education, and identification of unmet and comorbid care needs.

## 2. Materials and Methods

### 2.1. Design

This study employed a qualitative phenomenological approach to explore in depth how patients with COPD and emergency department (ED) medical officers and nurses perceive and make sense of COPD presentations within acute care settings, with particular focus on the role of nurses. A phenomenological design [22] was considered appropriate because it enables examination of lived experiences, meanings, and interpretations of care encounters that are not readily captured through quantitative measures or administrative datasets. Rather than measuring clinical outcomes, the study sought to understand how participants experience, interpret, and attribute meaning to nursing assessment, coordination, and support during acute exacerbations.

Phenomenology aims to foreground the subjective realities of participants and illuminate how everyday practices are experienced within their social and clinical contexts [22]. This perspective aligns closely with nursing inquiry, where relational, interpersonal, and contextual dimensions of care are central. By focusing on lived experience, the study sought to identify not only what occurs during COPD presentations, but how these events are perceived by patients and staff and how nursing actions shape those experiences. Reporting adhered to Standards for Reporting Qualitative Research (SRQR) to ensure transparency and methodological rigour [23].

### 2.2. Inclusion and/or Exclusion Criteria

Patient inclusion criteria were adults aged 18 years or older; a confirmed clinical diagnosis of COPD; recent ED presentation for an acute exacerbation; and capacity to participate in an in-depth interview. Staff inclusion criteria included nurses and other clinicians with direct experience managing COPD patients in the ED.

Participants were excluded if cognitive impairment, acute illness, or language barriers limited their ability to reflect on and articulate their experiences. Clinical judgement by treating staff determined fitness for participation. These criteria ensured participants could meaningfully describe their lived experiences while maintaining safety.

### 2.3. Study Setting and Recruitment

Participants were recruited using purposive, non-probabilistic sampling across three public hospital emergency departments located in Southern Queensland, Australia (Gold Coast University Hospital, Toowoomba Hospital, and Ipswich Hospital).

A total of 31 individuals were approached to participate in the study. Of these, 25 participants consented and completed interviews (response rate 81%). Six individuals declined participation due to illness severity (3), time constraints (2), or personal preference (1).

Recruitment continued until experiential data saturation was reached, defined as the point at which no new meanings or interpretive insights were emerging from interviews [24].

A narrative participant flow is outlined below:

- Thirty-one individuals approached;
- Twenty-five consented and interviewed;
- Six declined participation;
- None withdrew after interview;
- None excluded after transcription.

Given the qualitative phenomenological design, no further exclusions occurred following consent. All interviews were included in analysis.

Consistent with phenomenological methodology, the study sought experiential depth rather than statistical representation. Nevertheless, key demographic and clinical characteristics are summarised in Table 1 to contextualise the findings.

Patient Participants (n = 9)

- Age range: 58–84 years (median age 72 years);
- Sex: 5 female, 4 male;
- All had a confirmed clinical diagnosis of COPD;
- All presented to the ED with acute exacerbation within the preceding admission;
- Five participants reported ≥2 ED presentations within the past 12 months;
- Three participants were on long-term home oxygen therapy;
- Two reported prior ICU admission for respiratory failure.

Detailed spirometric classification and GOLD staging were not consistently accessible at the time of interview and were therefore not reported, as the study focused on lived experience rather than clinical severity categorisation.

Staff Participants (n = 16)

Registered Nurses (n = 9):

Clinical experience range: 4–22 years.

ED-specific experience range: 3–18 years.

Two held Clinical Nurse Consultant roles.

Medical Officers (n = 7):

Four Emergency Physicians.

Three Respiratory Physicians.

Experience range: 6–25 years.

Patients were approached during their ED presentation or subsequent inpatient admission by a member of the clinical team and provided with verbal and written information about the study. Staff participants were invited through departmental communication and professional networks. Interested individuals contacted the research team directly to minimise perceived coercion. Recruitment occurred progressively until experiential data saturation was reached, defined as the point at which no new meanings or perspectives emerged during interviews.

Participation was voluntary and unrelated to clinical care or employment status. Written informed consent was obtained prior to data collection.

### 2.4. Data Collection

Data were collected through individual semi-structured interviews designed to elicit rich narrative accounts of participants’ lived experiences as per Table 2. Interviews were conducted face-to-face or via secure videoconferencing according to participant preference and were held in private settings including hospital offices, meeting rooms, or participants’ homes to maximise comfort and confidentiality.

Interviews lasted between 40 and 60 min and were conducted by an experienced nurse researcher trained in qualitative interviewing. Questions encouraged participants to describe their experiences in their own words, focusing on perceptions of COPD presentations, interactions with nursing staff, assessment processes, discharge planning, and unmet needs. Probes were used to explore feelings, meanings, and interpretations rather than factual recall alone.

Field notes were recorded to capture contextual and non-verbal cues, such as pauses, emotional responses, or changes in tone, supporting interpretive depth. All interviews were audio-recorded and transcribed verbatim. Transcripts were returned to participants for verification (member checking), with no substantive amendments requested.

### 2.5. Data Analysis

Data analysis followed an inductive, interpretivist phenomenological epistemological position, using reflexive thematic analysis [25] to explore participants’ lived experiences of nursing care and COPD management. This interpretive stance assumes that knowledge is co-constructed through participants’ accounts and the researchers’ analytic engagement, and that meanings are contextually and socially situated. This approach was selected for its capacity to support in-depth interpretive engagement with experiential accounts while remaining flexible and consistent with a phenomenological focus on meaning-making. Researchers engaged in repeated reading of transcripts to achieve immersion and familiarity with participants’ accounts. Line-by-line coding was conducted to identify significant statements and experiential meanings derived inductively from the data, related to nursing care and COPD management without imposing pre-existing theoretical frameworks.

Using NVivo, codes were clustered into meaning units and progressively synthesised into broader experiential themes that captured shared patterns across participants through an iterative process of comparison, reflection, and refinement, while preserving the nuance of individual experiences. One researcher led the initial coding and theme development. A second and third researcher engaged critically with the developing analysis through reflexive dialogue rather than independent coding for agreement. This collaborative engagement involved discussion of analytic decisions, examination of alternative interpretations, and critical questioning of theme boundaries and coherence, thereby supporting theme refinement rather than resolution of discrepancies or verification of accuracy, in line with reflexive thematic analysis principles.

Coding, theme development, and data organisation were supported using qualitative data analysis software to assist with systematic data management and auditability. Analytic memos and an audit trail were maintained throughout the analytic process to document reflexive insights, interpretive decisions, and the evolution of themes, supporting transparency, confirmability, and methodological coherence.

### 2.6. Ethical Considerations

Ethical approval was obtained from the Queensland Health Human Research Ethics Committee (HREC/17/QGC/249). Written informed consent was obtained from all participants. Data were de-identified and stored securely. Participants were advised of their right to withdraw at any time without consequence.

### 2.7. Rigour and Reflexivity

Rigour was supported through adherence to SRQR [23] guidelines, member checking, peer debriefing, and maintenance of a detailed audit trail. Reflexivity was actively practised throughout. One researcher led the thematic analysis, while a second and third researcher engaged critically with the analysis through reflexive dialogue. They reviewed codes and themes, challenged interpretations, and supported refinement of the thematic structure by offering alternative perspectives and identifying areas requiring clarification or strengthening, thereby enhancing analytic depth and coherence rather than seeking consensus or verification.

Trustworthiness was established using the criteria of credibility, dependability, confirmability, transferability, and authenticity as described by Lincoln and Guba.

Credibility was supported through immersion in the data, iterative analysis, and inclusion of patient, nurse and medical officer perspectives. Verbatim quotations are used to demonstrate alignment between data and themes.

Dependability was ensured through a transparent and structured analytic process, supported by an audit trail documenting coding and theme development.

Confirmability was achieved through reflexive practices and grounding interpretations in participant data, supported by illustrative quotes.

Transferability was addressed by providing sufficient contextual detail to enable readers to assess applicability to other settings.

Authenticity was maintained by representing diverse participant perspectives, capturing the complexity of experiences within emergency COPD care [26].

## 3. Results

### 3.1. Overview of Themes

Analysis of interview transcripts revealed six interrelated themes that collectively describe how nurses function as the central integrators of care for patients presenting to the emergency department (ED) with acute exacerbations of COPD. Across participant accounts, nursing practice was characterised by continuous bedside presence, holistic assessment, relational engagement, and coordination of services that extended beyond the immediate episode of care.

Rather than operating solely within a biomedical model of acute intervention, nurses were shown to enact a form of clinical sense-making, integrating physiological indicators with patients’ lived experiences to inform decision-making.

While medical care was often described as episodic and diagnosis-focused, nursing care was consistently experienced as sustained, person-centred, and preventative in orientation. Participants positioned nurses as the clinicians who stabilised physiological crises, interpreted clinical complexity within patients’ everyday contexts, provided education to support self-management, coordinated transitions across services, and built therapeutic relationships that fostered trust and safety.

Importantly, these themes extend beyond description to demonstrate how nursing practice functions as a cohesive force within fragmented systems of care, actively mediating between acute management, patient experience, and continuity across services.

At the same time, nurses were described as working within organisational constraints that frequently limited their ability to deliver comprehensive preventative care.

This highlights a tension between the recognised scope of nursing practice and the structural conditions that shape what can be enacted within the ED.

#### 3.1.1. Theme 1: Nursing Within a “Crisis First” Model of Care

This theme describes nursing care delivered within a crisis-focused emergency model, where patients with COPD were commonly encountered at points of acute physiological deterioration. As illustrated in Table 3 (continuous bedside surveillance during acute deterioration), nurses were experienced as the constant bedside presence, providing minute-to-minute monitoring, rapid respiratory intervention, and emotional containment during episodes of severe breathlessness. Calming strategies and reassurance were integral to care, as panic was understood to directly worsen respiratory distress. Care was frequently reactive, with patients arriving late in the illness trajectory or deteriorating rapidly in the emergency department, requiring intensive intervention. This positioned nurses as both technical responders and relational anchors within a system primarily oriented toward rescue rather than early or preventative care.

Taken together, this theme demonstrates how emergency nursing practice was both constrained and defined by a crisis-focused model of care. Nurses were experienced as stabilising figures who held patients through moments of acute vulnerability, yet this relational and technical labour occurred within a system that prioritised rescue over anticipatory or preventative care.

**Accounts emphasised**: the central role of continuous nursing presence, respiratory support, and emotional regulation during episodes of acute respiratory distress:

We’re the ones who are actually there minute to minute—watching their breathing, adjusting the oxygen, noticing when they start to panic (RN2). When I couldn’t breathe, it was the nurse who was there the whole time. She didn’t leave (P3). You don’t really remember who the doctor was, but you remember the nurse because they’re the ones holding your hand (P5). They talk you through it—slow your breathing down (P1). It’s not just the oxygen. It’s calming them down, so they don’t spiral (RN4). Half of it is reassurance. If they panic, their breathing gets worse (RN6).

Patients frequently deteriorated prior to admission to specialist units, requiring rapid intervention and intensive nursing care. They just get straight onto BiPAP usually and they’re just pushing everything they can into them. So we start that weaning process as soon as we get them (RN6). A lot of them will come in and start failing while they’re in ED (RN3). During winter we have a CNC that goes down to ED and starts overseeing what is happening with the patient in resus … gets the respiratory consultants in there quicker (RN4). Sometimes I feel like we wish we’d seen them earlier rather than later. We see them when they’re crashed (D6). They’re so sick that they need one nurse to care for them (RN2).

**Interpretation**

This foregrounds how nurses simultaneously managed immediate survival and bearing witness to repeated late-stage deterioration, setting the context for the following theme, which explores the consequences of this model for continuity, workload, and moral tension in nursing practice.

#### 3.1.2. Theme 2: Holistic Assessment and Translation of Complexity

This theme captures how nurses framed their role as moving beyond biomedical parameters to interpret the person’s broader capacity to cope and remain safe. As reflected in Table 3 (holistic risk assessment beyond physiological metrics; nursing-led synthesis and communication of contextual risk), nurses consistently contrasted numerical indicators such as oxygen saturation with patients’ functional ability, social context, and lived realities. Assessment extended to questions about who supported patients at home, whether they could cook, shower, or live safely alone, and how they were managing day to day—factors perceived as central to clinical decision-making yet often overlooked by others.

Patients recognised this holistic focus, describing nurses as the only clinicians who asked about their lives beyond the immediate presentation. Nurses interpreted subtle functional changes—such as an inability to shower—as clinically significant “red flags,” even when physiological measures appeared stable.

**Accounts emphasised**: nurses’ holistic assessment of patients’ functional capacity, social context, and safety beyond physiological measures alone:

Doctors see numbers, nurses see the person (RN1). We’re asking what they’re normally like, not just what their sats are (RN5). The nurse asked me who helps me at home. No one else asked that (P4). She wanted to know if I could cook for myself or if I lived alone (P2).

Participants described nurses as considering the broader context of patients’ lives and capacity to cope after discharge. They think about the whole picture, not just the lungs (P1). They’re the ones asking if you feel safe going home (RN2). An 80-year-old who lives alone is very different to a 60-year-old with a partner at home—that matters when you’re deciding what’s safe (RN7).

Nurses reported identifying deterioration through functional decline and reduced coping, even when objective measures appeared stable. They might be oxygenating fine, but they tell you they couldn’t shower today—that’s a red flag (RN6). Sometimes it’s not that they’re crashing medically—it’s that they’re not coping anymore (RN3). The nurse wanted to know how I manage at home, not just what brought me in (P6). You can have someone with okay numbers but everything else is falling apart—that’s what we’re flagging (RN4).

**Interpretation**

This reflects nursing judgement as interpretive and relational, integrating lived experience with clinical observation. Within the emergency context, nurses positioned themselves as identifying risk not only through deterioration, but through signs that patients were no longer coping, reframing safety as a social and functional concern rather than a purely medical one.

#### 3.1.3. Theme 3: Education and Care Coordination as Preventative Nursing Work

This theme highlights persistent gaps in COPD self-management education and continuity of care. As outlined in Table 3 (education as a preventative nursing intervention; care coordination and system navigation), many patients report uncertainty about how to recognise or respond to exacerbations and rely on guesswork to decide when to seek emergency care. Nurses acknowledged that education and referral were often limited in the emergency department, contributing to repeated presentations. In response, nurses described assuming a central coordinating role, providing tailored education, reviewing inhaler technique, developing action plans, and facilitating multidisciplinary referrals. Readmissions were commonly interpreted as indicators of unmet support needs and systemic shortcomings, rather than individual patient non-compliance, reinforcing nursing work as pivotal in preventing recurrent crisis-driven care.

**Accounts emphasised**: gaps in education and discharge preparation, alongside the critical role of nurses in supporting self-management, care coordination, and reducing avoidable readmissions:

No one ever really explained what to do when it flares up (P2). I just guess when to come in (P6). Nurses acknowledged limitations in emergency care education and follow-up. We probably don’t do enough education in ED or refer for that matter (RN3). I know many have rebounded to the ED with the same compliance issues from last time (RN4).

Participants described how targeted nursing education and coordination promoted improved self-management. When the respiratory nurse showed me my inhaler properly, it changed everything (P7). The nurse organised physio, social work, everything (P5). If nurses didn’t chase things up, nothing would happen (RN1).

Nurses viewed education and continuity as core components of their role. Our job is around COPD action plans, the expert patients, self-management strategies (RN8). We do all of that on a daily basis through home visits and nurse-led clinics (RN9). Review of inhaler technique … making sure they can actually use those inhalers properly (RN5). If they’ve just been chucked an action plan on the way out the door … they won’t use it (RN2). If someone’s discharged and readmitted within 28 days, we need to look at what we missed (RN6).

**Interpretation**

Overall, this theme illustrates how gaps in education and continuity of care leave patients unprepared to manage exacerbations, positioning nurses—consistent with Table 3—as key agents of prevention and coordination. Nursing work extended beyond acute care to address systemic shortcomings, reframing recurrent presentations as failures of support rather than patient behaviour.

#### 3.1.4. Theme 4: Relational Care and Therapeutic Connection

This theme highlights the relational nature of nursing care and its influence on patients’ sense of safety, trust, and emotional regulation. In line with Table 3 (integration of emotional containment with physiological care; relational continuity for frequent presenters), patients described nurses as engaging with them “as a human,” fostering connection even during short emergency department stays. Nurses were experienced as a consistent presence, recognising patients across repeat presentations and holding relational knowledge that extended beyond the immediate clinical episode.

Trust emerged as central to care, shaping both emotional and physiological responses. Nurses described how calm, relational engagement helped settle anxiety and, in turn, stabilise breathing. This relational trust was also understood as enabling earlier help-seeking and disclosure of symptoms, with patients more likely to report deterioration sooner when they felt known and believed. Nursing care was thus experienced as therapeutic in itself, linking emotional safety, physiological stability, and timely access to care.

**Accounts emphasised**: the relational and emotional dimensions of nursing care, with trust, continuity, and calming presence described as central to patients’ sense of safety and engagement with care:

They talk to you like a human, not a number (P1). You feel safe with them (P5). Nurses are the ones you actually connect with (P3). Doctors come and go, but the nurses are the ones there the whole time (P6).

Nurses described deliberately fostering relationships, even during brief encounters, and viewed trust as clinically consequential. We build relationships even in short stays (RN2). Trust makes people come in earlier instead of waiting too long (RN4). If they trust you, they tell you earlier when something’s wrong (RN5).

Participants also linked nurses’ emotional regulation to patients’ physiological stability. If the nurse is calm, you calm down too (P7). Once they settle, their breathing settles. It’s connected (RN8). Continuity of care further strengthened this trust, with patients noting, They recognise you when you come back. They know your story already (P9).

**Interpretation**

This theme highlights how therapeutic nursing relationships foster trust, emotional safety, and physiological stability in acute COPD care. Nurses’ consistent presence and calm, relational engagement helped patients feel safe, disclose symptoms earlier, and regulate anxiety, positioning trust as a key enabler of timely care and improved respiratory stability.

#### 3.1.5. Theme 5: Nurses as Sentinels for Undiagnosed Comorbidities—Detecting OSA Risk

This theme highlights concerns that sleep-disordered breathing, particularly obstructive sleep apnoea (OSA), was under-recognised and under-addressed in patients presenting with COPD exacerbations. As reflected in Table 3 (opportunistic screening for comorbidities), medical officers and nurses described frequent suspicion of undiagnosed OSA, yet acknowledged that time pressures, lack of screening, and absent referral pathways in the emergency department meant these concerns were rarely acted upon. While simple screening tools were known, they were often deprioritised in the acute setting.

Nurses identified sleep as an important but overlooked aspect of assessment, noting that patients were more likely to disclose sleep-related symptoms to nursing staff during holistic conversations about daily functioning. Patients’ descriptions of waking gasping for air reinforced the potential contribution of sleep-disordered breathing to fatigue and breathlessness.

**Accounts emphasised**: possible under-recognition of obstructive sleep apnoea (OSA) among patients presenting with respiratory distress, alongside missed opportunities for screening and referral within emergency care pathways:

Some of these patients may be under-diagnosed with sleep apnoea (D1). My concern is they may have OSA, and we draft no referral (D2). Clinicians acknowledged that nurses were well positioned to recognise indicators of sleep-related breathing disorders. We are in a position to at least assess for OSA (RN6). We aren’t using the simple OSA screening tools … we’re time poor (RN4).

Sleep disturbance frequently emerged through holistic nursing assessment rather than targeted diagnostic processes. When you ask how they’re managing day to day, sleep always comes into it (RN3). They open up more to nurses about things like sleep (RN7). Patients also described symptoms consistent with sleep-disordered breathing, with one reporting, I often wake up gasping for air (P2).

Despite clinical suspicion, participants described the absence of clear referral pathways from the emergency department. Even if we suspect it, there’s no clear ED pathway to refer them (RN2). Clinicians noted that failing to address sleep apnoea risk may contribute to ongoing symptoms and repeated presentations: If OSA is driving fatigue and breathlessness, we’re missing a big piece (D6).

**Interpretation**

Nurses play a role as sentinels for hidden physiological risk. Overall, participants expressed concern that failing to identify and address OSA represented a missed opportunity to understand and manage an important driver of symptoms, contributing to ongoing morbidity beyond the immediate crisis.

#### 3.1.6. Theme 6: System Pressures and Constraints on Optimal Nursing Care

This theme captures how time pressure and throughput demands in the emergency department constrained opportunities for education, discharge planning, and preventative care for people with COPD. In keeping with Table 3 (structural support for preventative nursing work), nurses and medical officers described a work environment dominated by urgency and rapid turnover, where immediate crisis management took precedence over longer-term interventions. As busyness increased, education and holistic discharge planning were often the first elements to be omitted, resulting in superficial preparation for discharge despite recognition of ongoing risk.

Participants expressed frustration at knowing what would support better outcomes but lacking the structural time and resources to implement it. Preventative care was framed as optional rather than integral, reinforcing a cycle in which patients were discharged with an expectation of likely re-presentation. This positioned nurses as working within a system that prioritised short-term resolution over sustainable management, limiting their capacity to translate clinical insight into meaningful preventative action.

**Accounts emphasised**: how time pressure and throughput priorities constrained education, discharge planning, and preventative care, despite clinicians recognising their importance:

There’s time pressure, but that’s exactly when they need education (RN5). We’re trying to get people out quickly, so the bigger picture gets missed (D3). You end up firefighting rather than fixing things long term (D6).

Participants described how busyness directly shaped care priorities, with education and planning frequently de-prioritised. Education is the first thing that gets dropped when it’s busy (RN4). Discharge planning becomes very superficial (RN7).

While nurses were perceived as committed to preventative and holistic care, systemic constraints limited what could be achieved in practice. Nurses are doing the right things, but the system doesn’t give them space to do it (D2). Preventative stuff is seen as a bonus, not core business (RN3). You know what would help, but you can’t always do it (RN8). We discharge them knowing they’ll probably be back (RN9).

**Interpretation**

This theme indicates that systemic time pressure and throughput imperatives constrain nurses’ capacity to deliver education and preventative care, despite clear recognition of their importance. As a result, emergency care becomes oriented toward short-term crisis management rather than sustainable support, normalising repeat presentations and limiting opportunities for meaningful intervention despite clinicians’ insight and intent.

## 4. Discussion

This study explored the lived experiences of patients, nurses and medical officers to better understand the role of nurses in managing COPD presentations within the emergency department. Across six interrelated themes, nurses emerged not merely as task-oriented responders but as the central integrators of acute stabilisation, holistic assessment, education, coordination, relational care, and early detection of unmet needs. Collectively, these findings suggest that while ED systems are structured around rapid physiological rescue, nursing practice inherently bridges the divide between acute crisis management and chronic disease care. This integrative role aligns with contemporary descriptions of emergency nursing as a complex, multi-dimensional practice requiring advanced clinical judgement, coordination, and person-centred responsiveness [17,18].

Consistent with existing epidemiological evidence demonstrating that COPD remains a major driver of avoidable ED utilisation [1,2,3], participants described presentations as frequently crisis-based rather than preventative. Theme 1 highlighted the centrality of nursing surveillance and bedside presence during acute deterioration. While guidelines emphasise rapid escalation and oxygen titration during exacerbations [4,5], including national recommendations from the COPD-X guidelines [5], our findings extend this understanding by demonstrating that emotional reassurance and therapeutic presence are perceived as equally important components of stabilisation. This is strongly supported by qualitative evidence showing that ED nurses actively deliver person-centred care through presence, reassurance, and relational engagement, particularly during acute distress [15]. Furthermore, studies of emergency nurses’ experiences during COVID-19 highlight the intensity of this bedside role, where nurses assumed continuous responsibility for both physiological and emotional patient needs under pressure [13,16]. This aligns with qualitative syntheses describing breathlessness as both physiological and existential distress [12], reinforcing the importance of nursing’s relational and psychosocial contributions in emergency respiratory care.

Theme 2 further illustrates how nurses interpret clinical complexity through holistic assessment. Participants consistently reported that nurses gathered contextual information about function, supports, and home circumstances that directly influenced disposition decisions. These findings complement evidence suggesting that fragmented discharge planning contributes to repeated presentations [8,11] and support calls for more person-centred, context-aware approaches to COPD management. Importantly, emergency nursing competency frameworks emphasise comprehensive assessment—including social and functional domains—as a core capability in delivering safe, effective care in complex environments [17]. Rather than being ancillary, holistic assessment appeared intrinsic to nursing practice and critical for safe transitions of care.

Education and coordination, identified in Theme 3, were similarly positioned as preventative nursing work. Although guidelines advocate structured action plans and self-management education [4], and are reinforced within COPD-X recommendations [5], many patients in this study described limited instruction. This gap mirrors prior reports of inconsistent guideline adherence within ED settings [7,8], including Australian evidence demonstrating variable compliance with COPD care bundles and evidence-based management practices [9,10]. Evidence from nurse-led transitional care and community respiratory programmes demonstrates that coordinated follow-up and education reduce readmissions and emergency use [6,7]. Our findings suggest that ED nurses are already undertaking these functions informally but lack protected time and structured processes to do so effectively. This is further reinforced by literature highlighting the coordination and leadership roles of ED nurses, particularly charge nurses, in facilitating patient flow, communication, and care continuity across the system [18].

Themes 4 and 6 highlight the relational and systemic dimensions of care. Patients consistently described nurses as the “face of care,” with trust and therapeutic connection influencing help-seeking behaviour and engagement. This reflects established evidence that person-centred care in emergency settings is enacted primarily through nursing interactions, where patients value being treated as individuals rather than diagnoses [15]. Such relational continuity has been identified as central to effective chronic disease management [11,12]. However, organisational pressures prioritising throughput limited opportunities for preventative care, echoing broader concerns identified in studies of ED nurses during high-demand periods, where competing priorities constrain the ability to deliver holistic, relationship-based care [13,16]. This tension suggests that system redesign, rather than individual effort alone, is required to fully realise the benefits of nursing practice.

A novel contribution of this study is Theme 5, which identified nurses as potential sentinels for detecting undiagnosed obstructive sleep apnoea (OSA) in COPD. Overlap syndrome is increasingly recognised as a contributor to recurrent exacerbations and hospital use [19,20,21], yet routine screening remains uncommon. Participants described symptoms consistent with sleep-disordered breathing that were rarely assessed, despite nurses being well placed to identify such risks through routine history-taking. Given the emphasis within COPD-X guidelines on comprehensive assessment and management of comorbidities [5], integration of simple tools such as STOP-Bang within nursing assessment may represent a feasible, low-cost strategy to address this gap and aligns with recommendations for earlier identification of comorbid contributors to instability. This also aligns with broader conceptions of emergency nursing as encompassing early recognition and escalation of risk beyond the presenting complaint [17].

Taken together, these findings reinforce the view that nurses occupy a pivotal position at the interface between emergency and chronic care. Consistent with contemporary emergency nursing literature, this positions nurses not only as responders but as key agents of integration within fragmented health systems [17,18]. Formalising nurse-led assessment, education, coordination, and screening processes may enhance continuity, reduce preventable presentations, and improve outcomes for people living with COPD.

### 4.1. Implications for Practice

Findings from this study highlight several actionable opportunities to strengthen COPD care within emergency department settings through targeted nursing interventions. Across themes, nurses were consistently positioned as the clinicians most frequently present at the bedside, conducting holistic assessments, providing education, coordinating services, and building therapeutic relationships. These functions align closely with evidence-based strategies known to reduce exacerbations and avoidable hospital use, suggesting that formalising and supporting existing nursing practices may offer a practical pathway to improving outcomes.

First, embedding structured nurse-led assessment frameworks within ED workflows may enhance early identification of clinical and social risks. Incorporating brief holistic checklists that capture functional status, home supports, medication adherence, and self-management confidence could support safer discharge planning and reduce preventable re-presentations. Second, standardising education processes—such as short inhaler technique reviews, written COPD action plans, and reinforcement of early symptom recognition—may address the knowledge gaps described by participants. Evidence from transitional care and community respiratory programmes indicates that even brief, structured education delivered by nurses can significantly reduce readmissions and improve patient confidence.

Third, the coordination role undertaken informally by nurses could be strengthened through formalised referral pathways to pulmonary rehabilitation, community nursing, and primary care services. Designating nurse case management or liaison functions within EDs may improve continuity across care settings and align with integrated models recommended in contemporary COPD guidelines. Fourth, the identification of undiagnosed obstructive sleep apnoea as a contributor to instability highlights an opportunity to incorporate simple screening tools, such as STOP-Bang or Epworth Sleepiness Scale assessments, into routine nursing practice. Given that screening relies primarily on history-taking, nurses are well positioned to initiate early detection and referral without additional technological resources.

Future research is now required to strengthen the evidence base generated by this qualitative study through the use of prospective and experimental designs. In particular, clinical trials or pragmatic intervention studies are needed to evaluate the impact of nurse-led assessment, education, and coordination strategies on objective outcomes such as oxygen therapy optimisation, timeliness of escalation, rates of ED re-presentation, length of stay, and patient-reported outcomes, including satisfaction and confidence in self-management. Linking specific nursing interventions to measurable clinical and patient-centred outcomes would provide robust evidence to support broader implementation and policy investment in nurse-led models of COPD care within emergency settings.

Collectively, these strategies suggest that investing in nurse-led, preventative, and coordination-focused models may represent a feasible and cost-effective approach to reducing avoidable ED presentations while enhancing patient-centred care.

### 4.2. Limitations

Several limitations should be considered when interpreting these findings. First, the study was conducted across three hospitals within a single regional health service, which may limit transferability to other healthcare contexts with different organisational structures, staffing models, or patient populations. Although participants represented nurses, medical officers, and patients, the sample size was modest and reflective of qualitative methodology; therefore, results are intended to provide depth of understanding rather than statistical generalisability.

Second, data relied on self-reported experiences and retrospective accounts, which may be subject to recall bias or influenced by recent events. Participants’ descriptions of care processes may not fully reflect all clinical practices occurring within the ED. Third, the principal investigator’s background as an experienced respiratory nurse may have influenced interpretation despite deliberate reflexive strategies. While this insider perspective enhanced contextual understanding, it also required careful bracketing of assumptions to minimise bias.

Additionally, interviews were conducted at single time points rather than longitudinally, limiting exploration of how experiences evolve across repeated presentations or over extended periods of disease progression. Finally, although obstructive sleep apnoea emerged as an important finding, the study did not include objective diagnostic data to confirm prevalence; therefore, conclusions relate to perceived risk and missed opportunities for screening rather than confirmed clinical diagnoses.

Despite these limitations, the study provides rich experiential insights into nursing practice within ED COPD care and offers transferable lessons relevant to similar acute healthcare settings.

## 5. Conclusions

This study provides an in-depth exploration of how nurses, patients, and clinicians experience the management of COPD presentations within emergency department settings. Findings demonstrate that nursing work extends well beyond task-based acute care and instead functions as the primary mechanism through which crisis stabilisation is connected to longer-term chronic disease management. Across all themes, nurses were consistently positioned as continuous bedside clinicians who monitored deterioration, interpreted complex biopsychosocial needs, coordinated services, provided education, and established therapeutic relationships that fostered safety and trust. These contributions were frequently described as essential yet often invisible within systems primarily oriented toward rapid throughput and episodic treatment.

Importantly, the study highlights how preventative and relational aspects of nursing practice—particularly education, discharge planning, and early identification of comorbid risks such as obstructive sleep apnoea—represent underutilised opportunities to reduce avoidable exacerbations and recurrent presentations. While nurses are well placed to lead these functions, organisational constraints currently limit their capacity to deliver comprehensive preventative care.

Taken together, the findings suggest that strengthening and formalising nurse-led assessment, coordination, and screening pathways may offer a practical and scalable strategy to improve continuity, safety, and outcomes for people living with COPD. Investment in structured nursing models within ED settings is therefore critical to achieving more integrated, person-centred respiratory care.

## Figures and Tables

**Table 1:** Participant types and coding table.

Code Prefix	Participant Type	Description	Number (n)	Individual Codes
P	Patient	Adults with COPD presenting to the ED	9	P1–P9
RN	Registered Nurse	ED/respiratory nurses	9	RN1–RN9
D	Doctor	Respiratory + ED physicians	7	D1–D7
Total			25	

**Table 2:** Interview questions for understanding nurses’ role in caring for people with COPD in the emergency department.

Intro for Participants: “We’d Like to Learn About Your Experiences and Views of How People with COPD Are Cared for in the Emergency Department. Please Answer Based on What You Have Seen or Experienced.”
When someone with COPD comes to the emergency department, how do staff check what is happening with their breathing or lungs (for example observations or tests)? How well do you think this works?
What kinds of treatments or support are usually given for COPD in the emergency department? What seems to help most, and what could be better?
Many people with COPD also have other health problems. What other conditions or issues make COPD harder to manage in the emergency department?
Are patients given advice, education, or a written action plan to help manage their COPD at home? How clearly is this explained?
Who is usually involved in caring for someone with COPD in the emergency department, and what role do nurses play in organising or supporting this care?
In your view, what are the main reasons people with COPD come to the emergency department, especially during flare-ups or worsening symptoms?
What usually happens when someone is ready to leave the emergency department? Are follow-up plans or referrals made to other services (such as GPs, clinics, or pulmonary rehabilitation), and how easy are these to access?
Are there things like time pressure, busy departments, or limited resources that affect the care people receive? What changes would improve care for people with COPD?

**Table 3:** Key nursing considerations for future emergency department management of COPD.

No.	Nursing Consideration	Evidence from Themes	Implications for ED COPD Management
1	Continuous bedside surveillance during acute deterioration	Theme 1	ED nursing models should formally recognise nurses as primary sentinels for early deterioration, escalation, and ventilatory support during COPD exacerbations.
2	Integration of emotional containment with physiological care	Themes 1 & 4	Anxiety management and therapeutic reassurance should be embedded as clinical interventions, recognising their impact on dyspnoea and oxygenation.
3	Holistic risk assessment beyond physiological metrics	Theme 2	ED COPD assessments should routinely incorporate functional status, social supports, and coping capacity to inform admission and discharge decisions.
4	Nursing-led synthesis and communication of contextual risk	Themes 2 & 6	Structured mechanisms are needed for nurses to formally communicate psychosocial and functional risk to the multidisciplinary team.
5	Education as a preventative nursing intervention	Theme 3	COPD education (inhaler technique, symptom recognition, action plans) should be prioritised as core ED nursing work rather than optional discharge activity.
6	Behavioural coaching to promote early help-seeking	Themes 3 & 4	Nursing education should focus on confidence, decision-making, and timely GP/community engagement to reduce avoidable ED re-presentations.
7	Care coordination and system navigation	Themes 3 & 6	ED nurses should be supported to initiate referrals (respiratory, community nursing, allied health) through streamlined pathways.
8	Relational continuity for frequent presenters	Theme 4	Models of care should value relational nursing continuity, recognising its role in trust, adherence, and earlier presentation during exacerbations.
9	Opportunistic screening for comorbidities (e.g., OSA)	Theme 5	Nurse-led screening for sleep disturbance and overlap syndromes should be incorporated into COPD assessments with clear referral pathways.
10	Structural support for preventative nursing work	Theme 6	ED systems must allocate protected time and pathways to support education, screening, and discharge planning as essential nursing functions.

## Data Availability

The data supporting the findings of this study consist of qualitative interview transcripts generated from patients and healthcare staff. Due to privacy, confidentiality, and ethical restrictions associated with human participant research, these data are not publicly available. De-identified data may be made available by the corresponding author upon reasonable request and subject to approval by the relevant Human Research Ethics Committee and institutional governance requirements.

## Public Involvement Statement

Patients were involved in this study as participants, contributing their lived experiences and perspectives through qualitative interviews exploring the management of COPD presentations to the emergency department. Patient insights directly informed the identification of themes related to care processes, discharge planning, service gaps, and opportunities for improvement in nurse-led and coordinated models of care. Patients were not involved in the design of the study, development of the research questions, data analysis, interpretation of findings, or manuscript preparation. No consumers or carers were engaged as research partners or advisors.

## Guidelines and Standards Statement

This manuscript was drafted in accordance with the Standards for Reporting Qualitative Research (SRQR) guidelines for qualitative research (26).

## Abbreviations

The following abbreviations are used in this manuscript:

COPD	Chronic Obstructive Pulmonary Disease
ED	Emergency Department
OSA	Obstructive Sleep Apnoea
